# Establishment and characterization of matched immortalized human frontal and occipital scalp dermal papilla cell lines from androgenetic alopecia

**DOI:** 10.1038/s41598-023-48942-4

**Published:** 2023-12-05

**Authors:** Mi Hee Kwack, Ons Ben Hamida, Min Kyu Kim, Moon Kyu Kim, Young Kwan Sung

**Affiliations:** 1 Department of Immunology, School of Medicine, Kyungpook National University, 680 Gukchaebosang-ro, Jung-gu, Daegu, 41944 Republic of Korea; 2 BK21 FOUR KNU Convergence Educational Program of Biomedical Sciences for Creative Future Talents, School of Medicine, Kyungpook National University, Daegu, Korea; 3 Hair Transplantation Center, Kyungpook National University Hospital, Daegu, Republic of Korea

**Keywords:** Cell biology, Stem cells

## Abstract

Androgenetic alopecia (AGA), also known as male pattern baldness, is a common hair loss condition influenced by genetic and hormonal factors. Variations in gene expression and androgen responsiveness have been observed between the frontal and occipital regions of AGA patients. However, obtaining and cultivating frontal hair follicles is challenging. Therefore, no matched frontal and occipital dermal papilla (DP) cell lines have been reported yet. This study aimed to establish matched immortalized human frontal and occipital scalp DP cell lines from AGA patients. Simian virus 40 large T antigen (SV40T-Ag) and human telomerase reverse transcriptase (hTERT) were introduced into primary human DP cells. The obtained cell lines were characterized by assessing their gene expression patterns, androgen receptor (AR) levels, and the presence of 5-alpha reductase (5αR). Additionally, we examined their response to dihydrotestosterone (DHT) and evaluated cell viability. The conditioned medium from the frontal DP cell line inhibited human hair follicle growth, leading to reduced keratinocyte proliferation and increased apoptosis. Furthermore, when the cells were cultured in a 3D environment mimicking in vivo conditions, the 3D cultured frontal DP cell line exhibited weaker sphere aggregation than the occipital DP cell line due to the increased expression of matrix metalloproteinase 1 (*MMP1*), *MMP3*, and *MMP9*. Additionally, the expression of DP signature genes was inhibited in the 3D cultured frontal DP cell line. These matched frontal and occipital DP cell lines hold significant potential as valuable resources for research on hair loss. Their establishment allows us to investigate the differences between frontal and occipital DP cells, contributing to a better understanding of the molecular mechanisms underlying AGA. Furthermore, these cell lines may be valuable for developing targeted therapeutic approaches for hair loss conditions.

## Introduction

Dermal papilla (DP) cells are specialized mesenchymal cells located at the base of hair follicles. They play a crucial role in hair growth and maintenance by interacting with surrounding cells, including keratinocytes and melanocytes^1–3^. Cultured DP cells have been widely used in hair growth and regeneration studies^4–6^, however, they display drastic morphological changes and lose their characteristic features. In addition, prolonged DP cell culturing leads to reduced growth rate and overall proliferative capacity. DP cells also have a limited lifespan in culture, with a finite number of divisions before they reach senescence or growth arrest^7–10^. We previously established an immortalized DP cell line by introducing the simian virus 40 large T antigen (SV40T-Ag) and human telomerase reverse transcriptase (hTERT) into occipital primary DP cells^11^.

Androgenetic alopecia (AGA), commonly known as male pattern baldness hair loss, is a hereditary condition primarily affecting the hair on the scalp. The condition is characterized by the progressive miniaturization of hair follicles, leading to thinner, shorter, and less pigmented hair, eventually resulting in hair loss^12^. Unlike DP cells in the occipital (O) region, those in the frontal (F) region of AGA depend on androgens, specifically dihydrotestosterone (DHT), a potent form of testosterone. F DP cells are more sensitive to the effects of DHT than O DP cells. They make the hair follicles in the frontal region more susceptible to miniaturization and eventual hair loss in individuals affected by AGA. Different gene expression profiles are observed in F and O DP cells, contributing to the differences in hair growth and responsiveness to androgens between these regions. Mainly, androgen receptor (*AR*) and 5-alpha reductase type 2 (*5αR2*) are differentially expressed between F and O DP cells, with F DP cells expressing higher levels of *AR* and *5αR2* than O DP cells. In F DP cells with high *AR* expression, DHT reacts with AR and contributes to the miniaturization of hair follicles and hair loss in AGA^12–18^.

Understanding these differences in *AR* and *5αR2* expression in different scalp regions is essential for developing targeted treatments that address hair loss in AGA while minimizing side effects in unaffected areas. It also provides valuable insights into the underlying mechanisms of AGA and the potential for developing personalized therapeutic approaches for individuals with this condition. However, obtaining matched F and O hair follicles from AGA patients and isolating and culturing them is challenging. As a result, establishing matched immortalized human F and O DP cell lines has been rarely reported in scientific literature. In this study, immortalized F and O DP cell lines were generated by co-transfection of SV40T-Ag and hTERT and characterized by evaluating the expression of DP markers and *AR* and *5αR2*, characteristic markers associated with AGA. These cell lines may provide insights into the pathogenesis of AGA and could potentially lead to the development of targeted therapies for hair loss.

## Results

### Establishment of matched immortalized human F and O scalp DP cell lines from androgenetic alopecia

**Figure 1:**
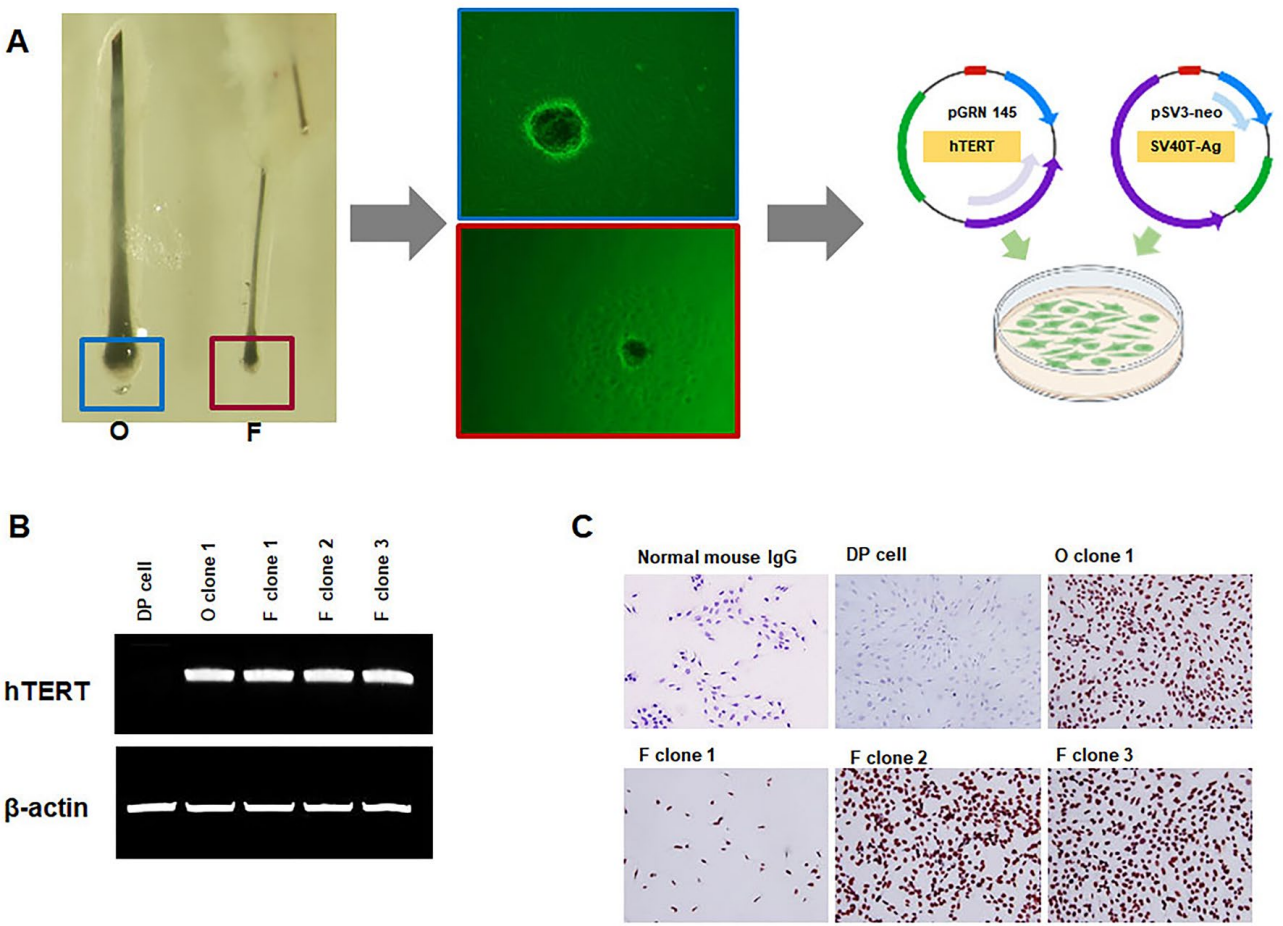
Establishment of matched frontal and occipital DP cells from AGA patients. (**A**) Illustration showing the establishment of F and O human DP cell lines from AGA patients. (**B**) Expression of *hTERT* in F and O cell lines. RT-PCR analysis was performed using equal amounts of total RNA from passage 2 DP cells (lane 1), O cells (lane 2), and F cells (lanes 3–5). (**C**) Expression of SV40T-Ag in F and O cell lines. SV40T-Ag was observed in the nucleus of O and F cell lines but not in primary human DP cells. Mouse IgG was used as a negative control.

A schematic diagram illustrating the establishment of F and O DP cells is shown in Fig. 1A. To establish a matched F and O DP cell line, we secured hair follicles undergoing hair transplantation surgery from matching frontal balding (F) scalp and occipital non-balding (O) specimens by punch biopsy (1 mm) from the same AGA patient and isolated and cultured the obtained DP cells. A pSV3 neo plasmid expressing *SV40T-Ag* and a pGRN145 plasmid expressing *hTERT* were co-transfected in F and O DP cells at passage 1 using a microporator. Neomycin- and hygromycin-resistant cells were selected, and the expression of *hTERT* and *SV40T-Ag* was observed by Reverse Transcription Polymerase Chain Reaction (RT-PCR) and immunocytochemical staining. The results indicated that matched F and O cell lines expressed *hTERT* and *SV40T-Ag* (Fig. 1B and C). *hTERT* and *SV40T-Ag* were not expressed in non-transfected primary DP cells.

### Expression of AGA-related genes in matched immortalized human F and O scalp DP cell lines

**Figure 2:**
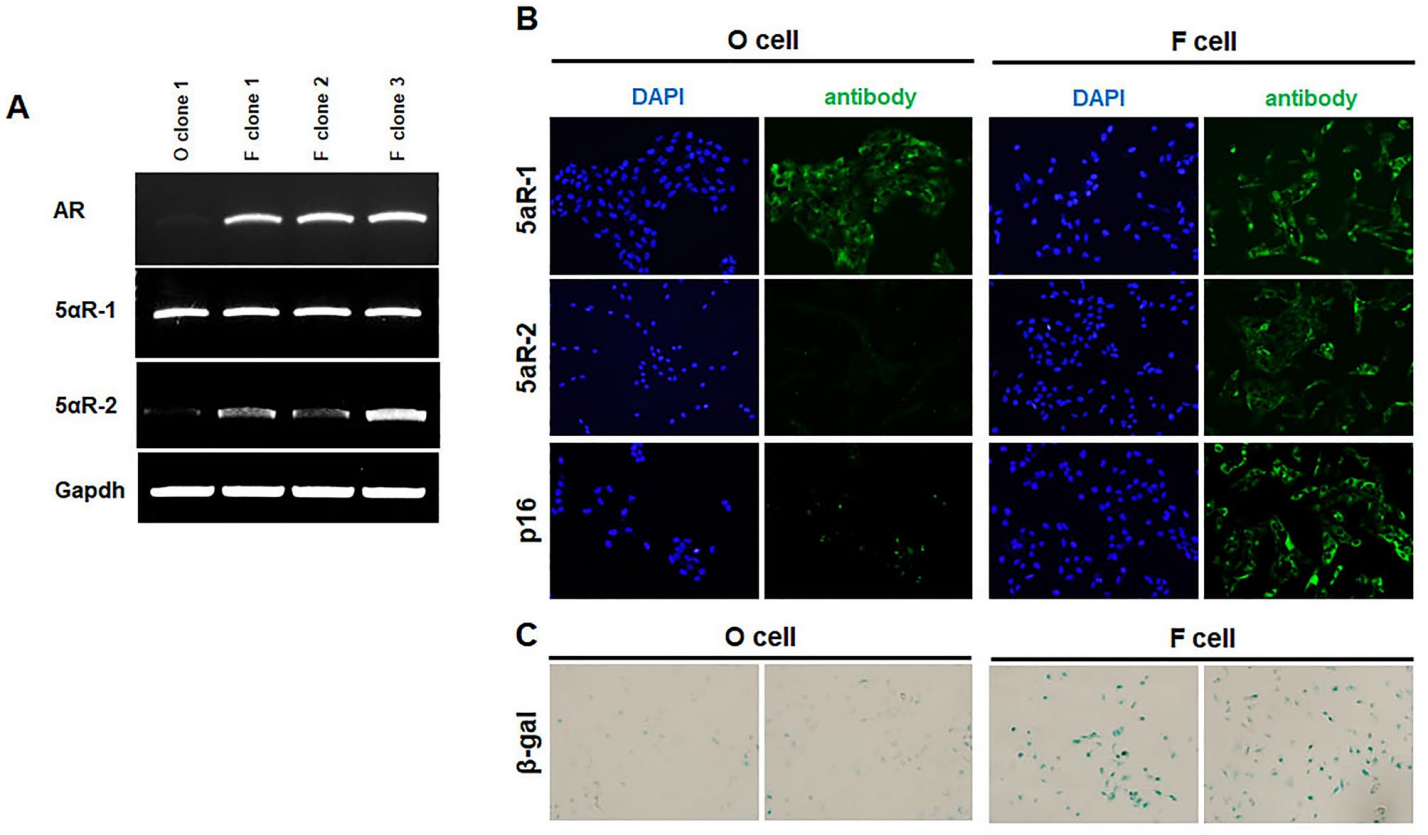
Characterization of matched frontal and occipital DP cell lines in AGA patients. (**A**) Total RNA was isolated from O and F cell lines at passage 35, and AGA-related genes were analyzed by RT-PCR. *GAPDH* was used as an internal control. (**B**) Matched F DP and O DP cell lines were immunostained with antibodies against 5αR-1, 5aR-2, and p16 (right panel). DAPI nuclear staining was also performed (light panel). (**C**) The F DP and O DP cell lines were subjected to β-gal staining.

Next, we characterized known AGA-related genes in these cell lines (Fig. 2A). *AR* and *5αR2* are signature genes of F DP cells, distinguishing them from O DP cells^18^. In addition, F DP cells express the senescence-associated *p16* and *β-gal*, unlike O DP cells^19^. As previously reported, *AR* and *5αR2* were highly expressed in established F DP cell lines but not in O DP cell lines (Fig. 2A). Matched F and O cell lines displaying different *AR* and *5αR2* expression have not been reported yet. In this study, among the three selected F cell clones, clone 3, with the highest *AR* and *5αR2* expression, was selected and analyzed in subsequent experiments. Additionally, senescence markers *p16* and *β-gal* were expressed higher in the F than in the O cell line (Fig. 2B and C).

### Expression of DP marker proteins in matched immortalized human F and O scalp DP cell lines

**Figure 3:**
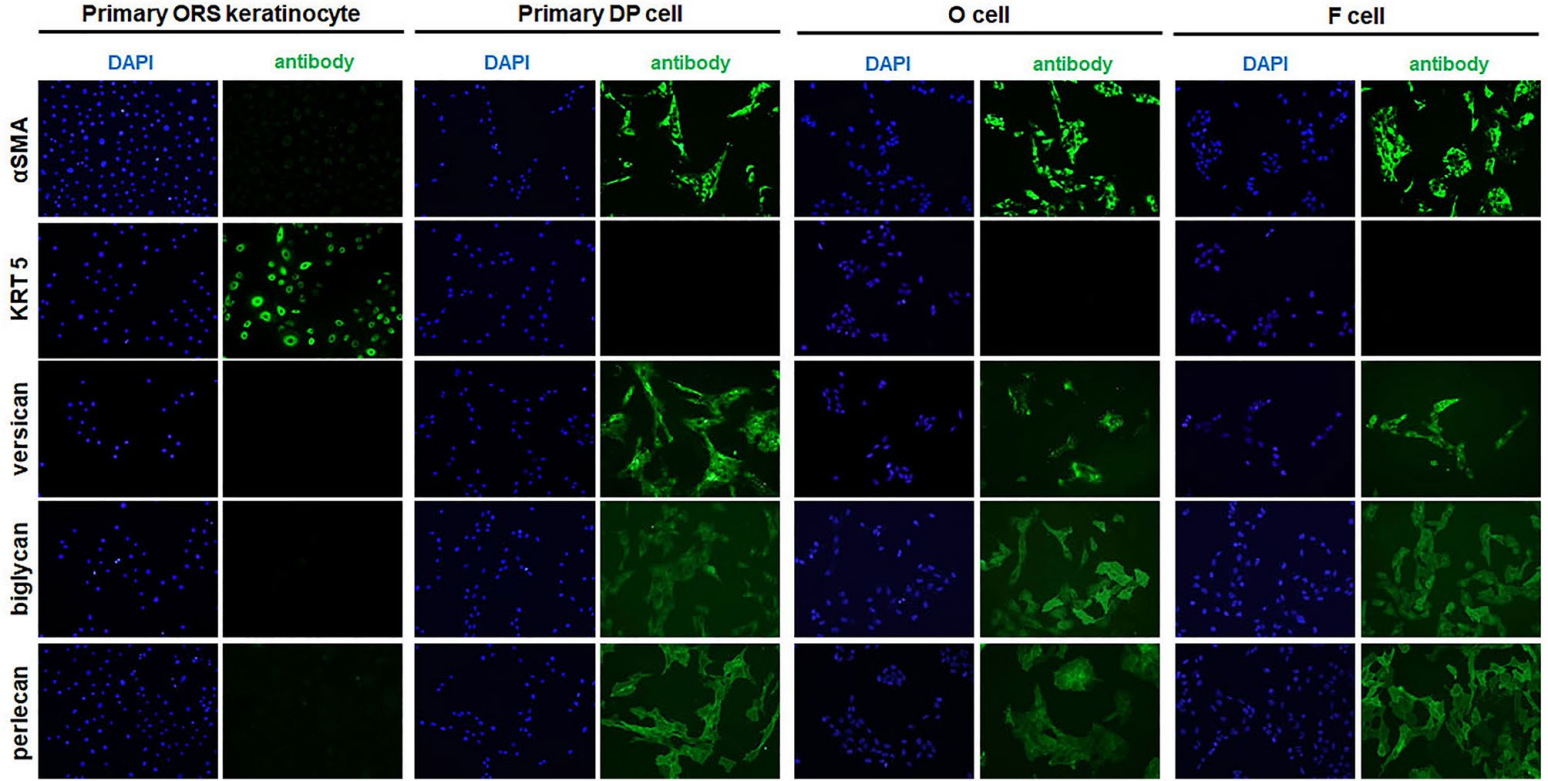
Expression of DP markers in matched frontal and occipital DP cell lines of AGA patients. Primary ORS keratinocyte (passage 2), primary DP cell (passage 2), and O and F DP cell lines were incubated with antibodies for αSMA, KRT5, versican, biglycan, and perlecan. DAPI nuclear staining was also performed (light panels). Staining of αSMA, versican, biglycan, and perlecan was observed in the primary DP cells and the O and F cell lines.

Next, we checked whether these newly established cell lines retained the properties of the primary DP cells (Fig. 3). Alpha smooth muscle actin (*α-SMA*) is a cultured DP cell marker that is expressed as highly in F and O cells as in primary DP cells but is not expressed in primary outer root sheath (ORS) keratinocytes. ALP activity is a helpful marker for identifying the location, shape, and size of DP and is correlated with the trichogenicity of DP cells^20–22^. ALP activity was expressed weakly in primary DP and O DP cell lines but not in F DP cell lines (Supplemental Fig. 1). On the other hand, cytokeratin 5 (*KRT5*), an epithelial cell marker, and von Willebrand factor, and endothelial cell marker, were not expressed in primary DP cells and O and F cell lines (Supplemental Fig. 2). Proteoglycans in the extracellular matrix (ECM) surrounding the hair follicle, including the dermal papilla region, are essential in maintaining the structure and function of the follicle^23^. The proteoglycans versican, biglycan, and perlecan are expressed in the O and F cell lines and the primary DP cells. These results indicate that the O and F cell lines maintain the properties of primary DP cells.

### Cell lines retain the properties of F and O DP cells in AGA

**Figure 4:**
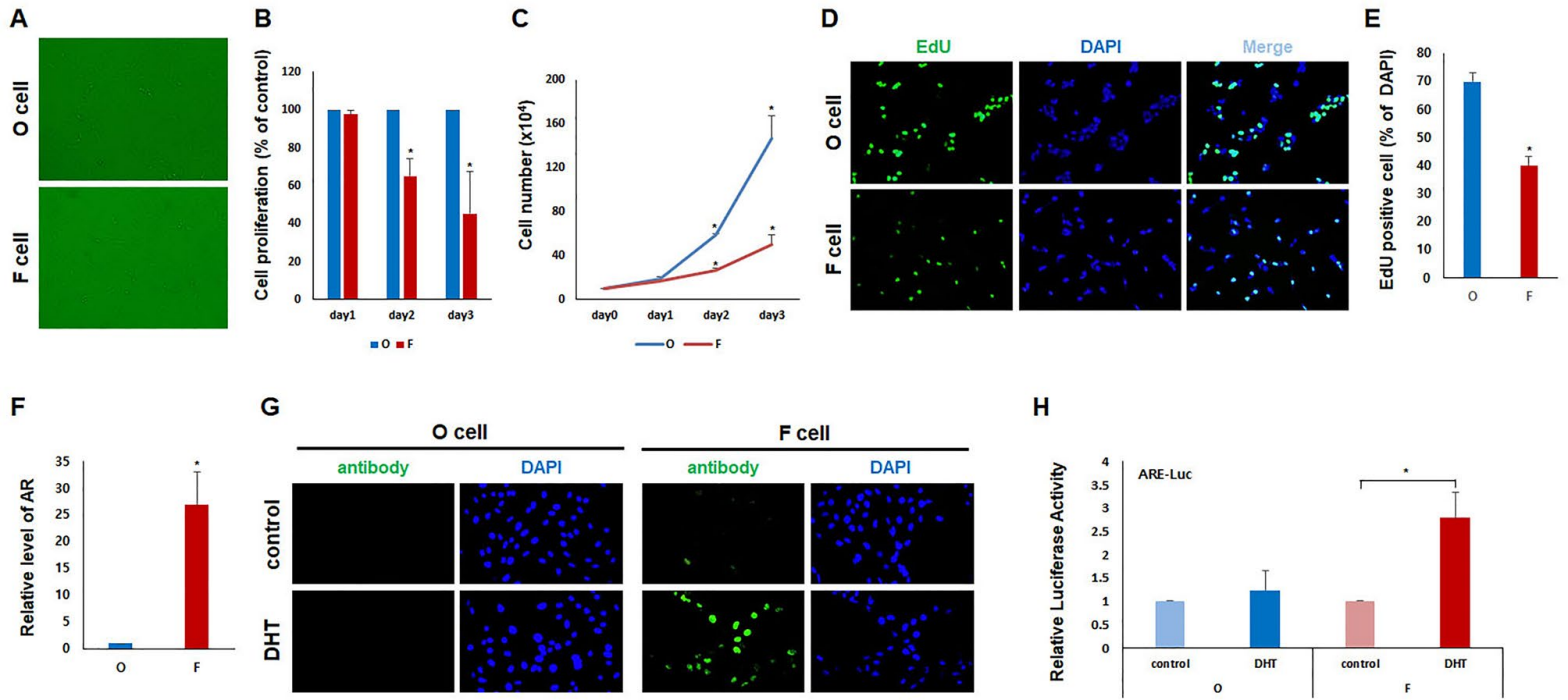
Characteristics of occipital and frontal DP cell lines. (**A**) Morphology of O DP and F DP cell lines. (**B**) The proliferation of O DP and F DP cell lines was analyzed by the CCK8 assay. Data are the mean ± SD from three independent experiments (*P < 0.05). (**C**) After seeding 10^5^ cells, cells were counted. Data are the mean ± SD from three independent experiments (*P < 0.05). (**D**) After incorporating EdU into the cells, proliferating cells were observed (left panel). Corresponding DAPI nuclear staining is also shown (middle panel). (**E**) EdU-positive proliferative cells were counted. Data are the mean ± SD from three independent experiments (*P < 0.05). (**F**) Relative *AR* levels were analyzed by real-time PCR. Data are the mean ± SD from three independent experiments (*P < 0.05). (**G**) Maintenance of responsiveness to DHT. Cells were seeded in chamber slides for 24 h, and cells were starved. After 24 h, cells treated with ethanol (control; top panels) or 100 nM DHT (bottom panels) for 3 h were immunostained with AR antibody. Accumulation of AR in the nucleus was observed by DHT treatment in F DP cell lines. (**H**) Cells were transfected with the pARE-luciferase plasmid and treated with or without 100 nM DHT for 24 h. Data are expressed as means ± SD of two determinations per experiment from three independent experiments (*P < 0.05).

F DP cells lack proliferative capacity, growing slower than O DP cells^19,24^. We verified the proliferation of established F and O DP cell lines by various methods. First of all, the morphology of the F and O DP cell lines at passage 50 generally displayed an elongated fibroblast cell shape, similar to the primary DP cells of the early passage (Fig. 4A). Cell proliferation assay using CCK-8 (Fig. 4B) indicated that the growth rate of F DP cell lines in vitro is much slower than that of O DP cells. Next, 10^5^ cells were seeded onto cultured plates, and cells were counted daily. The growth rate of the F DP cell line was significantly slower than that of the O DP cell line, confirming the results of the CCK-8 assay (Fig. 4C). EdU assay was performed to detect and quantify live cell proliferation. The most accurate method to measure DNA proliferation is directly measuring DNA synthesis. The O DP cells were 69.8% EdU-positive against 40.1% of the F DP cells, and the number of proliferating F DP cells was low (Figs. 4D and E, Supplemental Fig. 3).

Based on previous reports^14,18^, we investigated whether immortalized F DP cells expressed *AR*, responded to DHT, and affected AR activity. AR is a key factor in male pattern hair loss^18,25,26^. Real time-PCR analysis indicated that *AR* was more expressed in the established F DP cell lines than in the O DP cell lines (Fig. 4F). Additionally, applying 100 nM of DHT, a potent male hormone, for 3 h translocated *AR* to the nucleus in F DP cell lines but not in O DP cell lines (Fig. 4G). To evaluate DHT-induced AR activity in immortalized cell lines, we transiently transfected the AR reporter plasmid. After 6 h, DHT significantly increased the AR transcriptional activity (Fig. 4H). However, based on the AR immunofluorescence results, AR activity did not increase in the O DP cell lines. These results indicate that the matched F and O DP cell lines retain primary DP cell properties and maintain the balding and non-balding characteristics of DP in AGA patients.

### Effect of conditioned medium of immortalized O and F DP cell lines on the hair follicle

**Figure 5:**
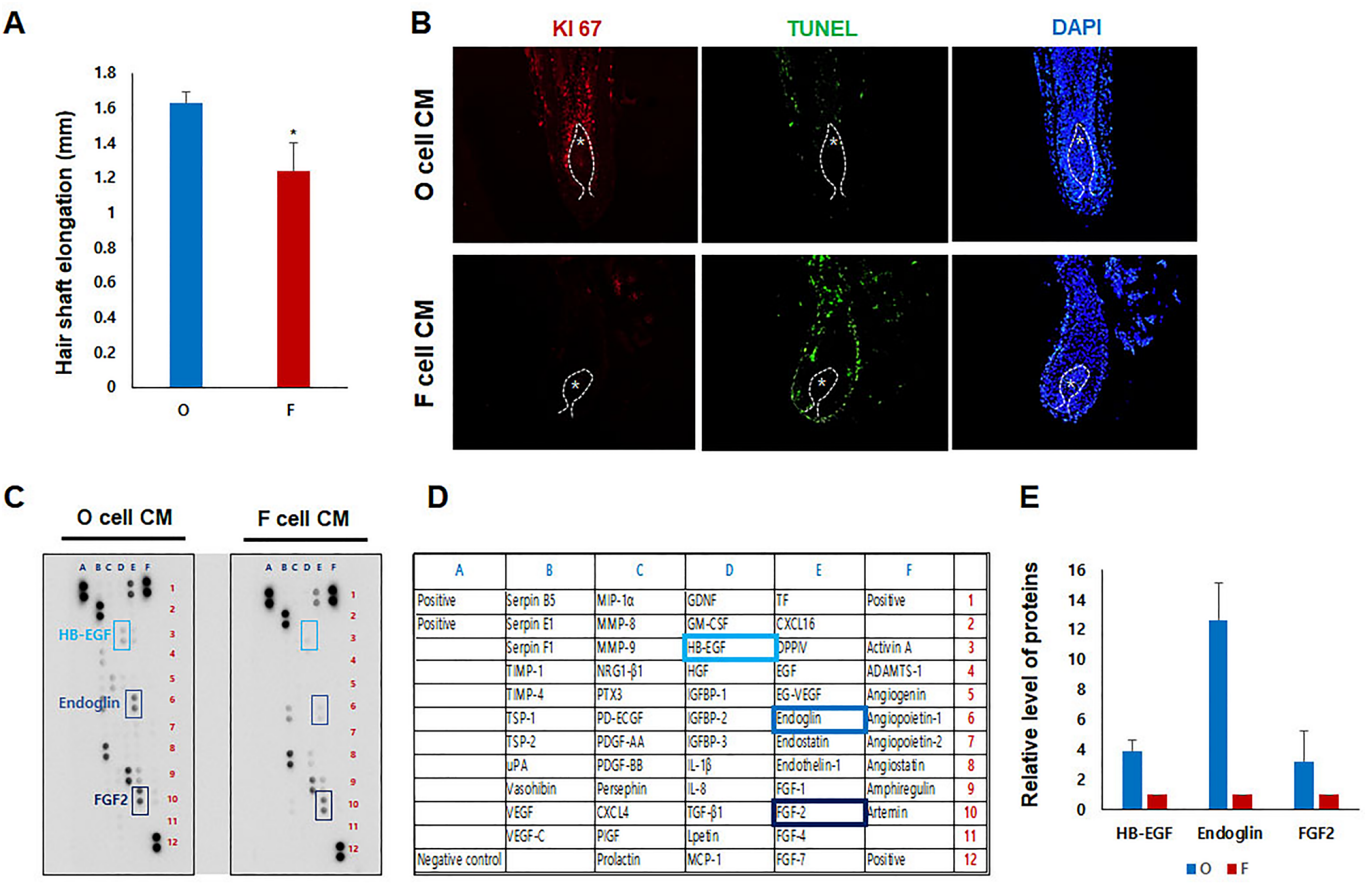
Effects of conditioned medium from occipital and frontal cell lines on human hair follicles. (**A**) Isolated human hair follicles were cultured for 6 days with CM from O and F DP cell lines, and the hair shaft elongation was measured. Values are expressed as means ± SD of seven measurements per experimental group, and experiments were repeated three times using hair follicles from three individuals (*P < 0.05). (**B**) After 3 days, follicles were immunostained with the Ki‐67 antibody (left panel) and underwent a TUNEL assay (middle panel). DAPI nuclear staining was also performed (right panel). White stars indicate DP in hair follicles. (**C**) The results of a proteome profiler array demonstrated the differential expression intensity of the secretory proteins in the CM of O and F DP cell lines. (**D**) List of proteins from the proteome profiler array. (**E**) The expression level of HB-EGF, Endoglin, and FGF2 was quantified using the Image-J program. Values are expressed as means ± SD of two spots.

Several previous reports suggested that F and O DP cells secreted different factors^24,27^. These factors have paracrine effects, influencing the surrounding keratinocytes and melanocytes, as well as autocrine effects, affecting the DP cells. First, we evaluated the effects of factors secreted by the immortalized O and F DP cell lines on human hair follicles. After concentrating the conditioned medium (CM) from the O and F DP cell lines ten times, human hair follicles were cultured, and the hair length was measured after 6 days. Consistent with previous reports^24,27^, we observed that the human hair elongation was inhibited in F DP CM-treated group compared to the O DP CM-treated (Fig. 5A). When the human hair follicle was treated with the F DP CM, the expression of Ki67, a proliferation marker, was significantly reduced compared to the O DP CM-treated group. Conversely, the number of TUNEL-positive cells, indicating apoptosis, increased in the F DP CM-treated hair follicles (Fig. 5B). Next, a proteome profiler array was performed to compare the factors secreted by the two cell lines (Fig. 5C). The secretion of heparin-binding EGF-like growth factor (HB-EGF), Endoglin, and fibroblast growth factor 2 (FGF2) was lower in the F DP CM-treated group, which displayed increased apoptosis and inhibited hair follicle length, than in the O DP CM-treated group (Fig. 5D and E).

### Cellular changes and differential expression in 3D-cultured O and F DP cell lines

**Figure 6:**
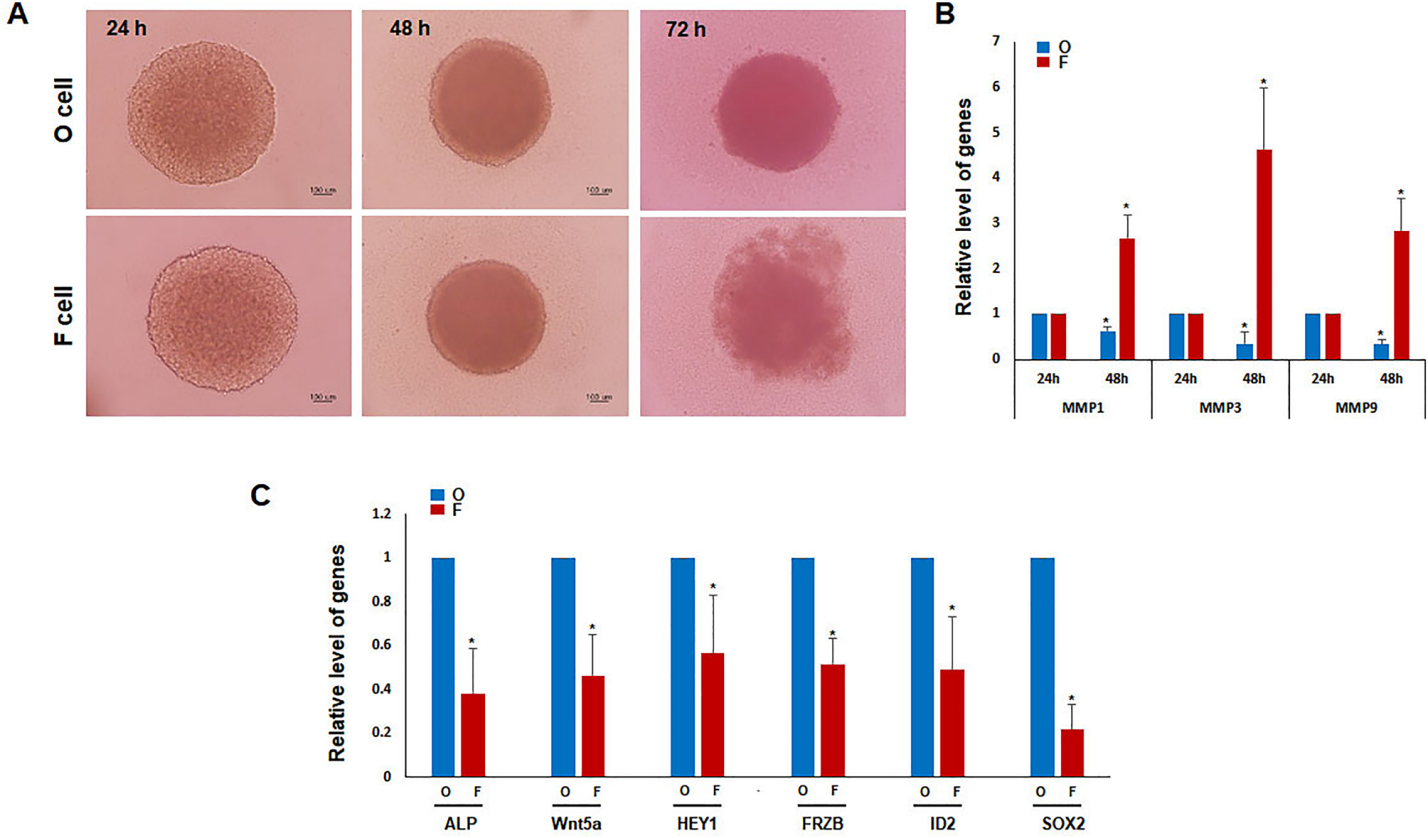
Differential gene expression in 3D-cultured occipital and frontal DP cell lines. (**A**) Representative image of the 3D culture using the 10^4^ O and F DP cell lines. Weakening of sphere aggregation is observed in the 3D culture of the F DP cell line after 72 h. (**B**) Expression levels of *MMP1*, *MMP3*, and *MMP9* were quantified by real-time PCR analysis in 3D-cultured F and O DP cell lines 24 and 48 h after sphere formation. Data are expressed as means ± SD of two determinations per experiment from three independent experiments (*P < 0.05). (**C**) Relative levels of representative DP signature genes were analyzed by real-time PCR in 3D-cultured F and O DP cell lines 48 h after sphere formation. Data are expressed as means ± SD of two determinations per experiment from three independent experiments (*P < 0.05).

Primary DP cells can express various genes during 3D culture and form hair follicles similar to the in vivo conditions^28–30^. O and F DP cell lines were established to observe the gene expression changes during 3D culture. Stable spheres were formed in both the O and F DP cell lines after 24 and 48 h. However, after a longer culture period (72 h), a significant outflow of DP cells was observed in the F DP spheres (Fig. 6A and Supplemental Fig. 4). Surprisingly, regarding O DP cells, the expression of matrix metalloproteinases *(MMP)1*, *MMP3*, and *MMP9* involved in aggregation and adhesion inhibition^31^, was reduced upon sphere formation. On the other hand, the F DP cell line displayed an increased expression of *MMP1*, *MMP3*, and *MMP9* after 24 and 48 h of 3D culture (Fig. 6B).

Next, the expression of DP signature genes was compared between the two cell lines. DP signature genes are highly expressed in human fresh DP^32^. Interestingly, the known DP signature genes, including *ALP*, *Wnt5a*, *HEY1*, *FRZB*, *ID2*, and *SOX2*, were expressed at lower levels in the F DP cells than in the O DP cells (Fig. 6C). Among the DP signature genes, *ALP*^22,29,32,33^ and *SOX2*^34,35^ are two trichogenes involved in hair follicle formation. These genes are essential in developing and regulating hair follicles, and their expression is crucial for properly functioning and maintaining hair follicle structures. Although the expression of DP signature genes is higher in the established O DP cells compared to F DP cells, new follicle formation was not observed (data not shown).

## Discussion

The hair follicle formation ability of cultured human DP cells is reduced, and their proliferative capacity has gradually diminished during sub-cultivation. As a result, a few research groups have attempted to establish immortalized DP cell lines to overcome these limitations and maintain the cell properties in long-term culture^1–11^. Recently, Ikeda et al.,^36^ has established a cell line capable of forming hair by transfecting human telomerase reverse transcriptase (*hTERT*) and B-cell-specific Moloney murine leukemia virus insertion region 1 (*BMI1*) into human scalp DP cells to avoid growth arrest. 

DP cells from AGA patients exhibit differences in cell proliferation capacity, gene and protein expression, and responsiveness to androgens depending on the hair follicle location along the scalp^12–14^. Therefore, matched immortalized F and O DP cell lines are required for male pattern balding researches, but frontal region hair follicles are difficult to obtain, isolate, and culture.

Based on previous reports^11^, we introduced *SV40T-Ag* and *hTERT* into matched O and F DP cells to establish immortalized cell lines and selected cell lines expressing both *hTERT* and *SV40T-Ag*, essential for achieving immortalization and maintaining cell proliferation capacity. To verify the differences between O and F DP cell lines in AGA, *AR* and *5αR2* expression was examined. 5αR has two isotypes in humans, 5αR1and 5αR2, with 50% homology in their amino acid sequence^37,38^. 5αR is an enzyme that converts testosterone into its more potent form, DHT. DHT binds to AR within the F DP cells of AGA patients and plays a significant role in the miniaturization of hair follicles by inducing the activation or repression of target genes^14,18^. Consistent with other studies *AR* and *5αR2* were weakly expressed in O DP cells while highly expressed in F DP cells. Among the three F DP cell lines studied, the one with the highest *AR* and *5αR2* expression was selected for further analysis. Additionally, *p16* and *β-gal*, characteristic markers of F DP cells^19^, were highly expressed in the selected cell line (Fig. 2). The maintenance of primary DP cell characteristics was verified in the immortalized O and F DP cell lines by examining the expression of several DP markers. The expression of α-SMA, versican, biglycan and perlecan was comparable between the primary DP cells and the immortalized O and F DP cell lines, indicating that these immortalized DP cell lines maintained some features of primary DP cells. However, the immortalized DP cell lines did not express the keratinocyte marker KRT5 and the endothelial cell marker VWF. This finding is consistent with the fact that DP cells are of mesenchymal origin and do not typically express keratinocyte-specific markers (Fig. 3). Overall, the verification of DP marker expression and *AR* and *5αR2* expression in the immortalized O and F DP cell lines supported their suitability as model systems for studying AGA and hair biology. F DP cells exhibited a slower cell proliferation rate than O DP cells in AGA and displayed a higher AR activity in response to DHT (Fig. 4). This study aimed to confirm whether the established F and O DP cell lines displayed these specific characteristics. The proliferation rate of the F DP cell line was significantly lower than that of the O DP cell line. Furthermore, the F DP cell line exhibited a higher AR activity against DHT than the O DP cell line, further supporting the previously reported findings. Furthermore, we observed the expression of AGA-related genes, cell proliferation, and DP signature genes in three different established O DP cell clones and F DP cells. These results were consistent with matched O and F DP cell lines (Supplemental Figs. 5, 6, 7 and 8).

Studies have identified several DP cell-secreted factors that influence hair growth and pigmentation. These factors include various growth factors, cytokines, and signaling molecules, such as Wnt proteins, fibroblast growth factors, transforming growth factor-beta (TGF-β), and platelet-derived growth factor (PDGF). These molecules can affect the surrounding cells’ proliferation, differentiation, and function. We studied the effect of factors secreted from the established O and F DP cell lines on human hair follicles. The results indicated that the F DP cell line exhibited a reduced expression of HB-EGF, Endoglin, and FGF2 compared to the O DP cell line. The CM of the F DP cell line reduced keratinocyte proliferation and increased the number of Tunnel-positive cells (Fig. 5). HB-EGF is a member of the EGF protein family, is expressed in DP cells, and promotes hair follicle development and maintenance through several mechanisms. It stimulates the proliferation of hair follicle cells, including matrix cells that produce the hair shaft, and regulates the regeneration and repair of the hair follicle^39–41^. Endoglin, also known as CD105, is a transmembrane protein involved in various cellular processes, including angiogenesis, tissue development, and stem cell regulation. Endoglin expression may influence the behavior and activity of these stem cells, affecting hair follicle cycling and maintenance^42–44^. FGF2, also known as basic fibroblast growth factor, is a member of the fibroblast growth factor family. FGF2 signaling is involved in hair follicle patterning and morphogenesis during embryonic development. It regulates the interaction between epithelial and DP cells, which are essential for the formation and growth of hair follicles, and promotes cell proliferation in the hair follicles, including matrix cells that produce the hair shaft^45–48^. The factors released by cells are critical in regulating hair follicle function and development but are reduced in established F DP cell lines (Fig. 5).

Regarding human DP cells, the expression of DP signature genes and trichogenes involved in hair follicle formation can be recovered during 3D cultivation. In this study, the F DP cell line did not form spheres properly compared to the O DP cell line, where sphere formation was achieved within 72 h of culture. The inability of F DP cells to form spheres properly was attributed to an increase in the expression of *MMP1*, *MMP3*, and *MMP9*, which led to the prevention of aggregation. Among those genes, *MMP1* wa upregulated in the DP cells of balding scalp areas compared to non-balding areas^49^. The increased expression of *MMP1* has led to increased enzymatic activity, which, in turn, resulted in the degradation of collagen and other ECM components in the hair follicles. This degradation weakens the structural support of the hair follicles, contributing to their miniaturization and eventual hair loss. The F DP cell line displayed a decreased expression of genes related to hair follicle formation. Consistent with a previous report^50^, the 3D F DP cell line did not display proper follicle formation, unlike the O DP cell line.

In this study, we established immortalized cell lines using matched O and F DP cells of AGA.

Using immortalized F and O DP cell lines is beneficial in research as it allows investigating cellular and molecular processes related to AGA in a controlled and reproducible environment and might contribute to treating and preventing hair loss.

## Materials and methods

### Isolation and culture of human hair follicle DP and ORS cells

Units of human hair follicles were obtained during hair transplantation from matching balding (frontal) and non-balding (occipital) scalps of patients by punch biopsy (1 mm). The Medical Ethical Committee of the Kyungpook National University Hospital approved this study (IRB Number KNUH 2021-09-006), and written consent was obtained from patients according to the Declaration of Helsinki Principles. DP and outer root sheath (ORS) keratinocytes were isolated as previously described^11^. Briefly DP were isolated from the bulbs of dissected hair follicles, transferred onto plastic dishes coated with bovine type 1 collagen, and cultured in Dulbecco's modified Eagle's medium (DMEM; Hyclone, Logan, UT, USA) supplemented with penicillin (100 U/ml), streptomycin (100 µg/ml), and 20% heat-inactivated fetal bovine serum (FBS) at 37 °C in a humidified atmosphere of 5% CO2. The explants were left for 7 days, and the medium was changed every 3 days. After primary DP cell outgrowth had become sub-confluent, cells were harvested with 0.25% trypsin/10 mM EDTA in Hank's balanced salt solution (HBSS) and were maintained in DMEM supplemented with 10% FBS. In the subsequent experiments, primary dermal papilla (DP) cells at passage 2 were used. The hair shaft and hair bulb region of the hair follicles (HFs) were removed to prevent contamination with other cells.

HF remaining after cutting the bulb were then placed in Dulbecco's modified Eagle's medium (DMEM) supplemented with 20% fetal bovine serum (FBS) in tissue culture dishes coated with Biocoat collagen type I (CORNING, Kennebunk, ME04043, USA). After 3 days of culture, the medium was replaced with keratinocyte growth medium, EpiLife (Gibco BRL, Gaithersburg, MD, USA), containing 1% antibiotic–antimycotic solution and 1% EpiLife defined growth supplement. Once the cells reached subconfluency, they were harvested using 0.25% trypsin/10 mM EDTA in phosphate-buffered saline (PBS) and maintained in EpiLife medium. Primary ORS cells from the second passage were used for the experiments in this study.

### Construction of immortalized F and O DP cells

F and O DP cells at passage 1 were transfected with a pSV3neo plasmid (ATCC; Manassas, VA, USA) carrying *SV40T-Ag* and neomycin resistance and a pGRN145 plasmid (ATCC) carrying *hTERT* and hygromycin resistance. Briefly, cells (5 × 10^5^) were transfected with 1 μg pSV3neo or pGRN145 as an internal control using Microporator (Invitrogen, Carlsbad, CA, USA; pulse voltage 1100, pulse width 50). After 48 h, immortalized cells were selected with 5 μg/ml hygromycin B (Thermo Fisher) and 5 μg/ml G-418 (Sigma). *SV40T-Ag* and *hTERT* expression was evaluated by immunocytochemistry and RT-PCR analysis, respectively. Immortalized frontal and occipital DP cell lines were cultured in Dulbecco’s modified Eagle’s medium (DMEM; Hyclone, Logan, UT, USA) with 10% heat-inactivated fetal bovine serum and were used for 35–50 passages in this study.

### Cell proliferation assay

Immortalized F and O DP cells were plated in 96-well plates (1,000 cells per well). Cells were measured 24, 48, and 72 h after seeding with a cell counting kit-8 (Donjindo, Kumamoto, Japan). The absorbance was measured using a microplate reader (Molecular Devices, Sunnyvale, CA, USA) at 450 nm. For direct counting methods, 10^5^ immortalized F and O DP cells were plated in 10 mm. After 24, 48, and 72 h, cells were harvested with 0.25% trypsin/10 mM EDTA in Hank’s balanced salt solution and counted. An EdU proliferation kit (Abcam) was used following the manufacturer’s protocol to evaluate DNA synthesis in live cells. Cells were cultured in a 96-well plate for 24 h, treated with 20 μM EdU, and cultured for 3 h. After activation using EdU Additive Solution Reaction Buffer, DNA proliferation was observed under a microscope.

### β-galactosidase staining

A β-galactosidase staining kit (Cell Signaling) was used to evaluate cell senescence. Briefly, after fixing at room temperature for 10 min, washing twice with PBS, and incubating overnight in β-galactosidase staining solution at 37 °C, senescence cells were observed in blue color under a microscope**.**

### RT-PCR

Total RNA was isolated using RNeasy Mini Kit (Qiagen, Hilden, Germany), and cDNA was synthesized from 3 μg total RNA using ImProm-II™ reverse transcriptase kit (Promega, Madison, WI, USA). PCR was performed using a Taq polymerase and forward and reverse primers, and the product was confirmed under UV light after electrophoresis in 1% agarose. Real-time PCR was performed using Step One Plus Real-Time PCR System (Applied Biosystems, Foster City, CA, USA) with 50 ng cDNA, 10 pM primers, and Power SYBRR Green premix (Applied Biosystems). The cycling conditions for amplification were: 95 °C for 10 min, 40 cycles at 95 °C for 15 s, and 60 °C for 60 s. Primer sequences are listed in Supplementary Table 1.

### Immunocytochemistry and immunofluorescence staining

For the immunocytochemistry of SV40T-Ag, immortalized F and O DP cells were seeded on 8-well slides (Nunc Lab-Tek, Roskilde, Denmark) at passage 4 after a 24-h treatment with hygromycin B and G418. Cells were fixed in 4% paraformaldehyde containing 0.1% Triton X-100 for 10 min at room temperature. After 3% H_2_O_2_ treatment for 30 min, blocking was performed in 5% normal donkey serum (Abcam, Cambridge, UK) for 1 h, and samples were incubated with antibodies against SV40T-Ag (1:100 dilution; Santa Cruz Biotechnology, Santa Cruz, CA, USA) at 4 °C overnight. Cells were washed 3 times with PBS and incubated with horseradish peroxidase-conjugated donkey anti-rabbit antibody for 1 h. After washing with PBS, color developed using AEC, and counterstaining was performed with hematoxylin.

For the immunofluorescence staining, cells were seeded, fixed, and blocked, as described above. Next, cells were incubated with antibodies against a-SMA (R&D Systems, Minneapolis, MN, USA), cytokeratin 1–3 (KRT 8; Chemicon, Temecula, CA, USA), versican (Seikaguka Corporation, Tokyo, Japan), biglycan (R&D Systems), perlecan (Zymed Laboratories, San Francisco, CA, USA), and AR (Abcam, Cambridge, UK) at 4 °C overnight. After washing three times with PBS, cells were incubated for 1 h at room temperature with Alexa Fluor 488-labeled donkey anti-rabbit or mouse secondary antibody (Molecular Probes, Eugene, OR, USA). Subsequently, slides were washed with PBS and counterstained with 4,6- diamidino-2-phenylindole (DAPI) for 10 min.

### CM of immortalized F and O DP cells and proteome profiler array

After seeding 10^6^ cells in a 10-mm plate, the medium was replaced with serum-free DMEM. After 24 h, the CM was harvested from F and O DP cells. The CM was concentrated using Amicon Ultra (Millipore) by centrifugation and filtration and applied to human hair follicles. After 6 days of CM treatment, the length of human hair follicles was measured. A proteome profiler array (R&D Systems, Minneapolis, MN, USA) was used to analyze growth factors displaying differences between immortalized Fal and O cells.

### TUNEL and Ki-67 staining

A TUNEL kit (EMD Millipore, Billerica, MA, USA) was used following the manufacturer’s protocol to evaluate apoptotic cells. In brief, cells were fixed in 1% paraformaldehyde for 10 min and post-fixed in ethanol-acetic acid (− 20 °C) for 5 min. Slides were washed with PBS, incubated with working strength TdT enzyme at 37 °C for 1 h, and incubated with digoxigenin fluorescein-conjugated antibody for 30 min. Next, cells were incubated with antibodies against Ki-67 (Millipore) at 4 °C overnight. After washing with PBS, Alexa Fluor 555-labeled donkey anti-mouse secondary antibody was incubated for 1 h at room temperature. The slides were counterstained with DAPI.

### 3D culture of immortalized F and O DP cells

The 3D cultured cells were harvested and re-seeded in a 96-well Hydro Cell plate (Nunc, Rochester, NY, USA). After 24, 48, and 72 h, pictures were taken under a microscope, and RNA was isolated.

### Statistical analysis

Data are expressed as means ± standard deviation (SD). ANOVA was used for statistical analysis of the data. *P* < 0.05 was considered statistically significant.

## Statement of Ethics

The study was conducted according to the Declaration of Helsinki Principles. Informed written consent was obtained from the patient. The Medical Ethical Committee of the Kyungpook National University Hospital (Daegu, Korea) approved all of the described studies (IRB Number KNUH 2021-09-006).

### Supplementary Information

Supplementary Figures.

Supplementary Table 1.

## Data Availability

All relevant data are included in the manuscript. Used datasets are available from the corresponding author on reasonable request.
