# Supplementary material for: Establishment and characterization of matched immortalized human frontal and occipital scalp dermal papilla cell lines from androgenetic alopecia
Source: Sci Rep. 2023 Dec 5;13:21421. doi: 10.1038/s41598-023-48942-4 (PMC10696020; doi:10.1038/s41598-023-48942-4)
Supplement: Supplementary file 1 — Supplementary Figures. [file 41598_2023_48942_MOESM1_ESM.pdf]

Supplement Figure 1

ALP activity

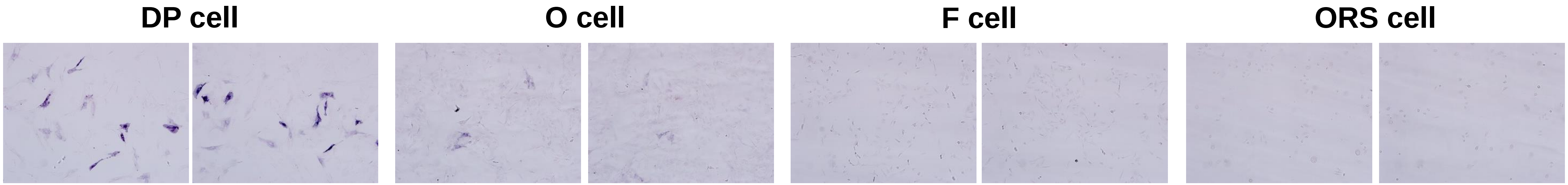

Primary DP cell (passage 2), O DP cell line (passage 45), F DP cell line (passage 45), primary ORS cell (passage 2) were assessed alkaline phosphatase (ALP) activity. ALP activity is detected in cultured primary DP cells and is weakly detected O DP cell line. However ALP activity is not detected in F DP cell line and ORS keratinocyte (ALP negative control).

Supplement Figure 2

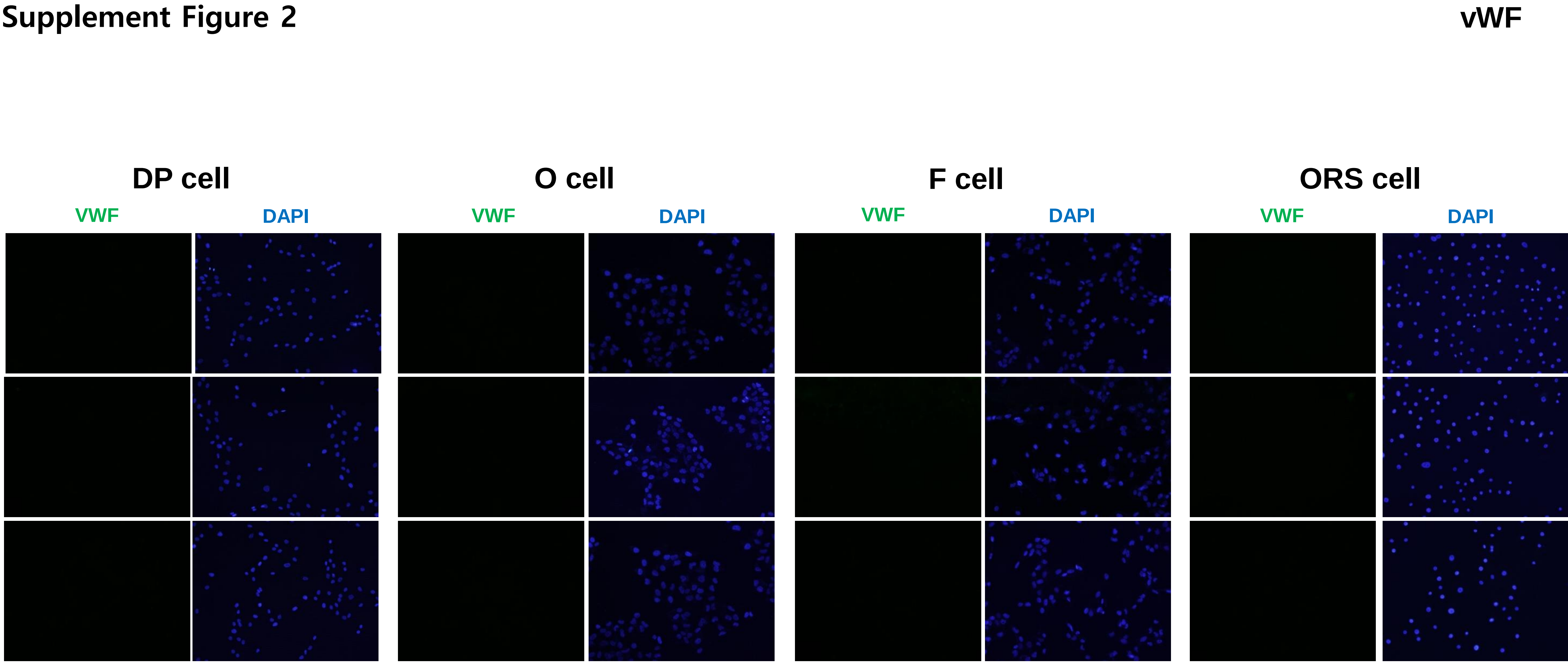

Primary DP cells (passage 2), O DP cell line (passage 45), F DP cell line (passage 45), primary ORS cells (passage 2) were assessed von Willebrand factor (vWF; endothelial cell marker). vWF is not expressed in primary DP cells, O and F DP cell lines and ORS keratinocyte.

Supplement Figure 3

EdU staining

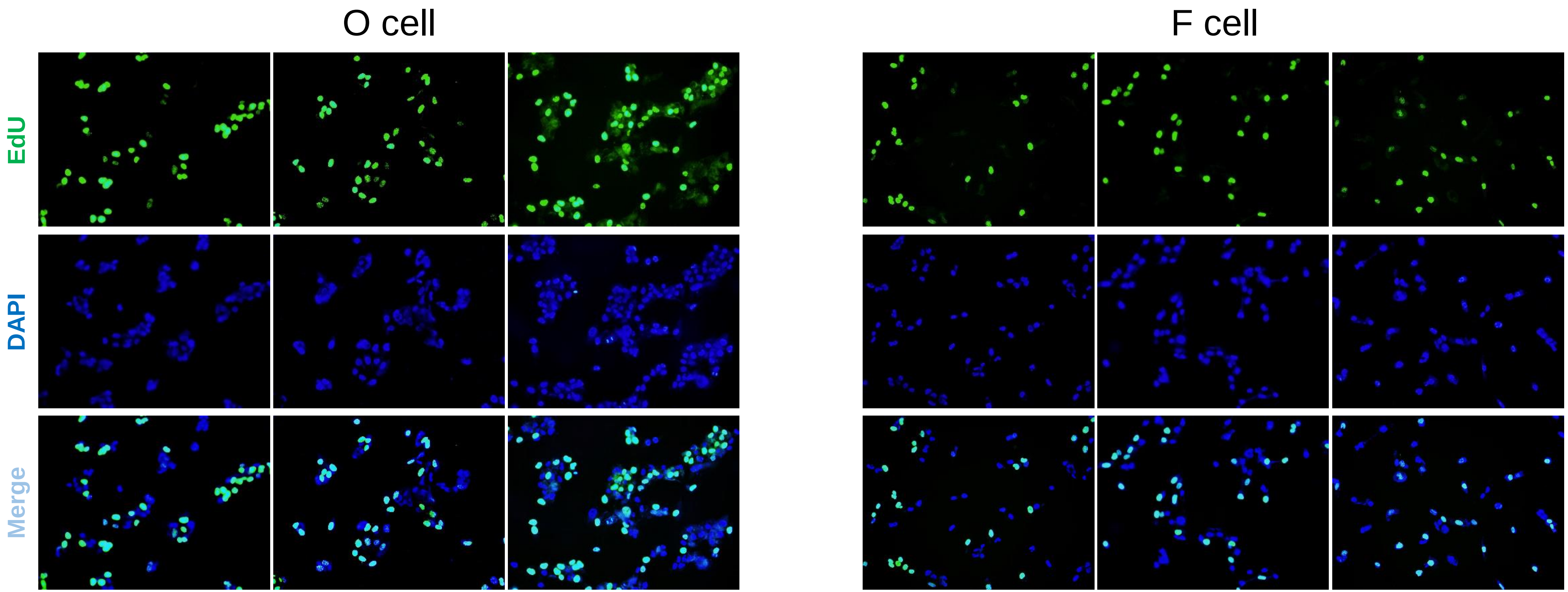

After 10^5 cells O and F DP cell lines seeding for 24h, cells were incorporated Edu. After incorporating EdU into the cells, proliferating cells were observed (upper panel). Corresponding DAPI nuclear staining is also shown (middle panel).

Supplement Figure 4

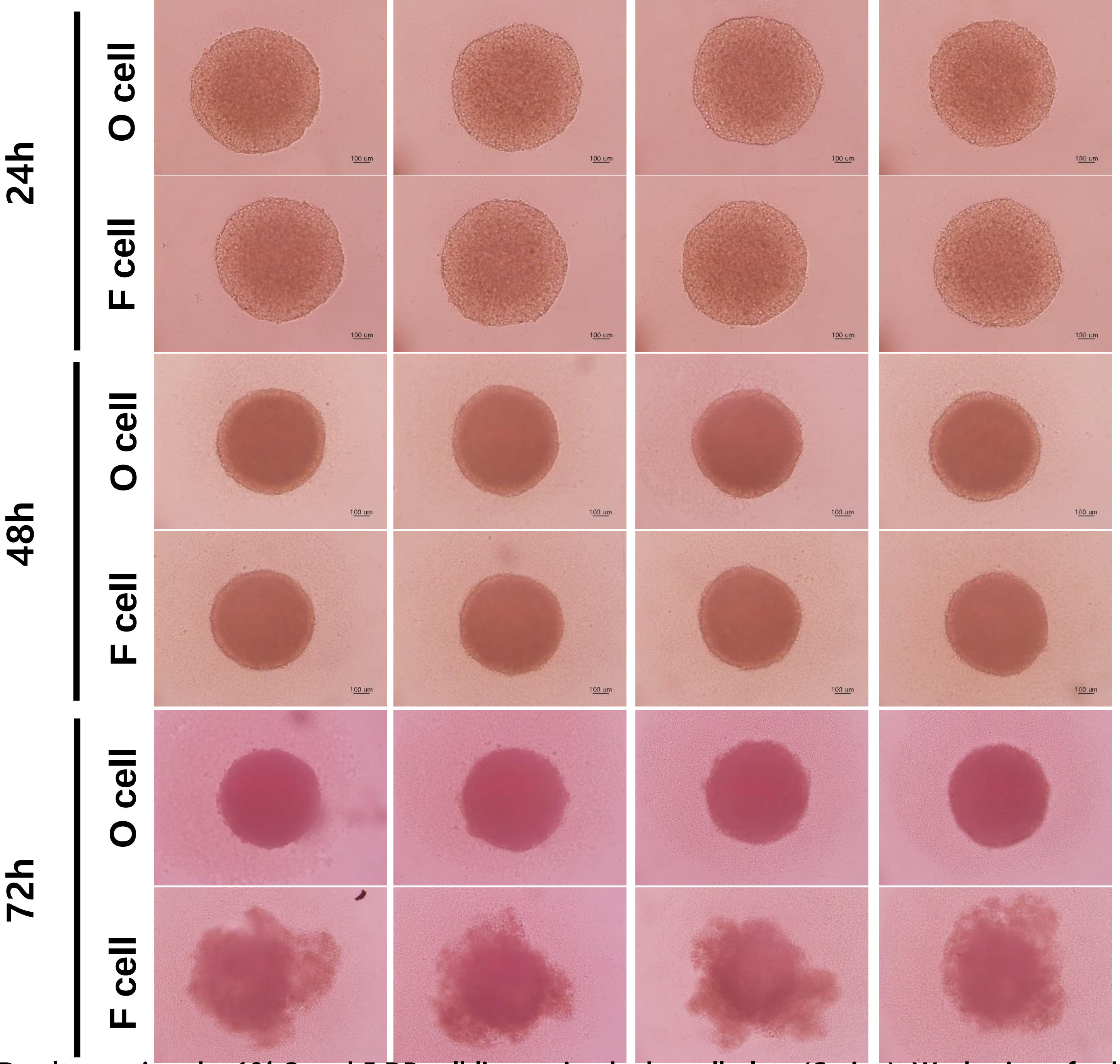

Representative image of the 3D culture using the 10^4 O and F DP cell lines using hydro cell plate (Coring). Weakening of sphere aggregation is observed in the 3D culture of the F DP cell line after 72 hours.

Supplement Figure 5

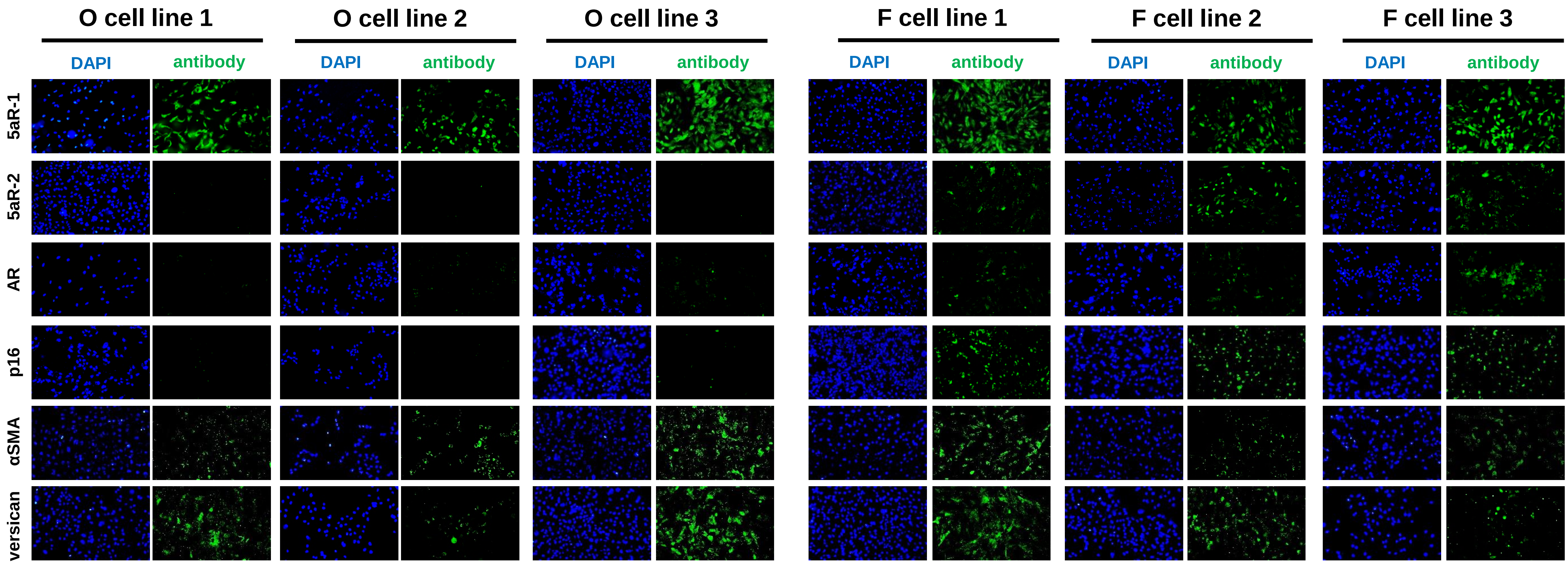

After 10^5 cells three types of O and F DP cell lines seeding for 24h, cells were assessed AR related proteins (5aR-1, 5aR-2, AR and p16) and cultured DP cells markers (aSMA, versican). 5aR-2, AR and p16 are not expressed O DP cell lines.

Supplement Figure 6

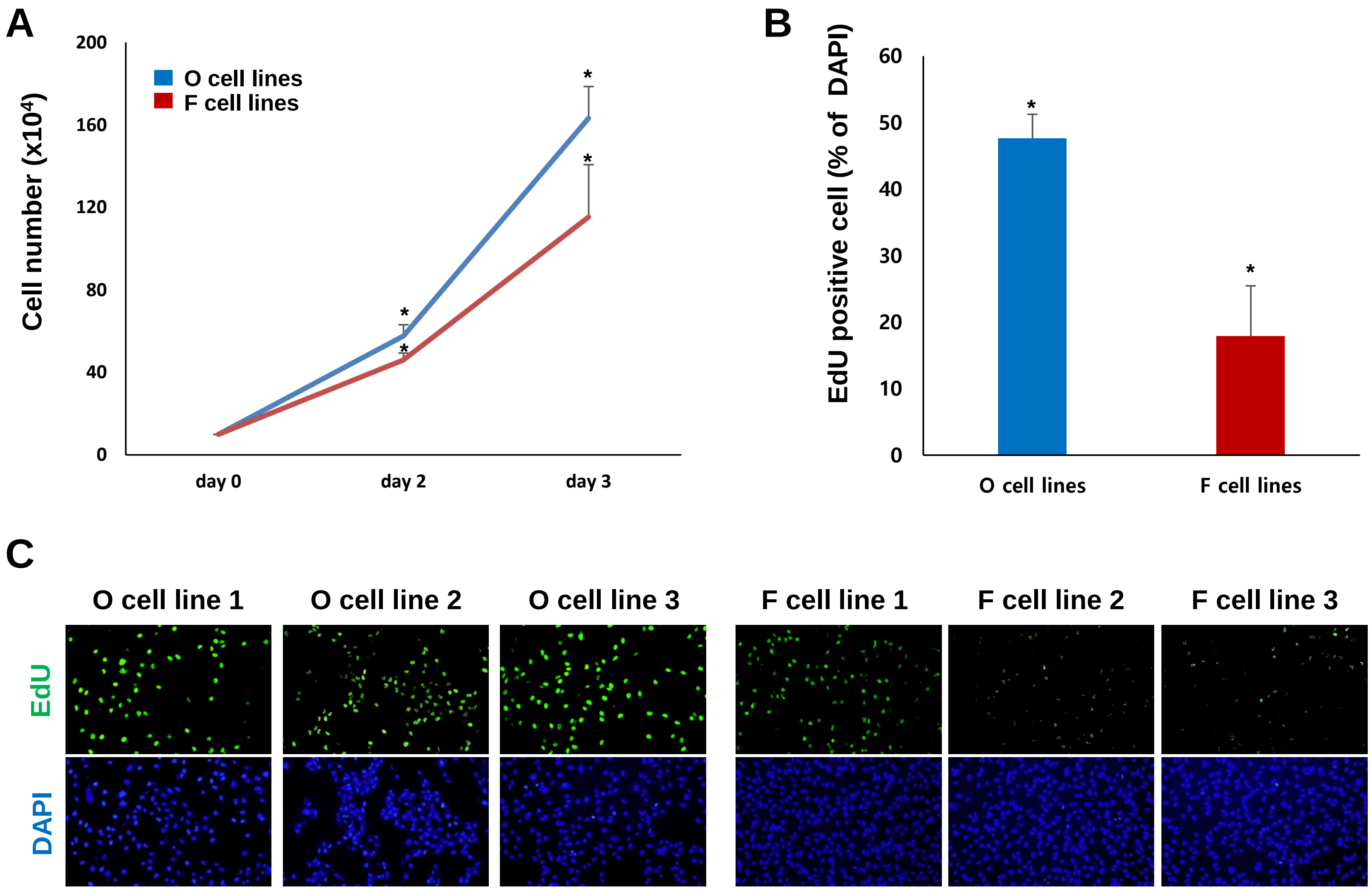

(A) After seeding 10^5 cells, three types of O DP cell lines and three types of F DP cell lines were counted. Data are the mean ± SD from three clones(**P* < 0.05). (B) EdU-positive proliferative cells were counted. Data are the mean ± SD from clones (**P* < 0.05). (C) After incorporating EdU into the cells, proliferating cells were observed (left panel). Corresponding DAPI nuclear staining is also shown (middle panel).

Supplement Figure 7

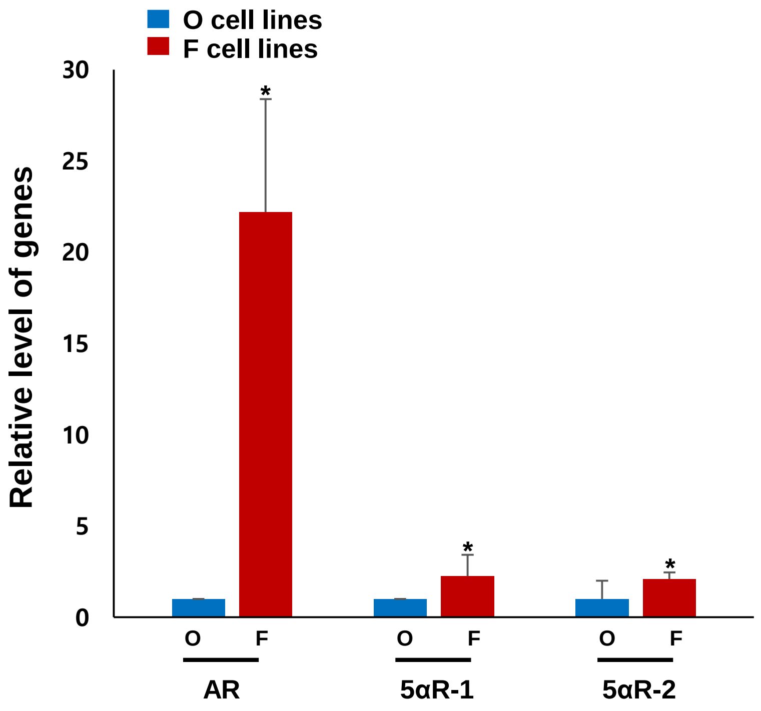

Relative AR, 5aR-1 and 5aR-2 levels were analyzed by real-time PCR. Data are the mean ± SD from three clones (*P < 0.05).

Supplement Figure 8

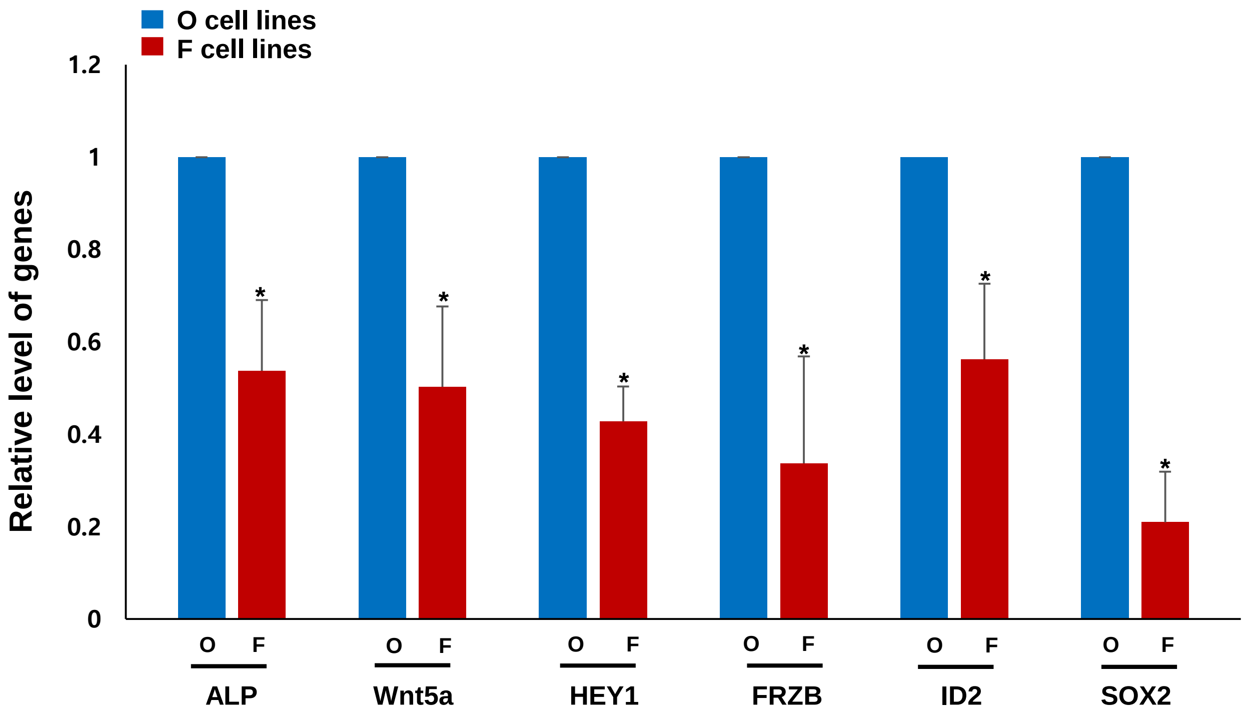

Relative levels of representative DP signature genes were analyzed by real-time PCR in 3D-cultured F and O DP cell lines (three clones) 48 hours after sphere formation. Data are expressed as means ± SD from three clones (*P < 0.05).
