# Supplementary material for: Establishment and characterization of matched immortalized human frontal and occipital scalp dermal papilla cell lines from androgenetic alopecia
Source: Sci Rep. 2023 Dec 5;13:21421. doi: 10.1038/s41598-023-48942-4 (PMC10696020; doi:10.1038/s41598-023-48942-4)
Supplement: Supplementary file 2 — Supplementary Table 1. [file 41598_2023_48942_MOESM2_ESM.docx]

Table Supplementary 1. PCR conditions and primers used in this study.

| Gene | Oligonucleotide primers | | Cycle  numbers | D | A | P |
| --- | --- | --- | --- | --- | --- | --- |
| ß-actin | GGGAAATCGTGCGTGACATT | GGAGTTGAAGGTAGTTTCGTG | 25 | 95 | 58 | 72 |
| GAPDH | AACTTTGGCATTGTGGAAGG | ACACATTGGGGGTAGGAACA | 23 | 95 | 60 | 72 |
| AR | GGTAAGGGAAGTAGGTGGAA | CCTTCTAGCCCTTTGGTGTA | 28 | 95 | 60 | 72 |
| 5aR-1 | CAATGGCGCTTCTCTATGG | TACACACAGCACCTGACACG | 28 | 95 | 60 | 72 |
| 5aR-2 | TGAGGTTACATGCTGCTTGC | TCCAATTACAAGCGTTCGG | 28 | 95 | 55 | 72 |
| hTERT | CGGAAGAGTGTCTGGAGCAA | GGATGAAGCGGAGTCTGGA | 28 | 95 | 58 | 72 |

denaturation, annealing, and polymerization temperature
